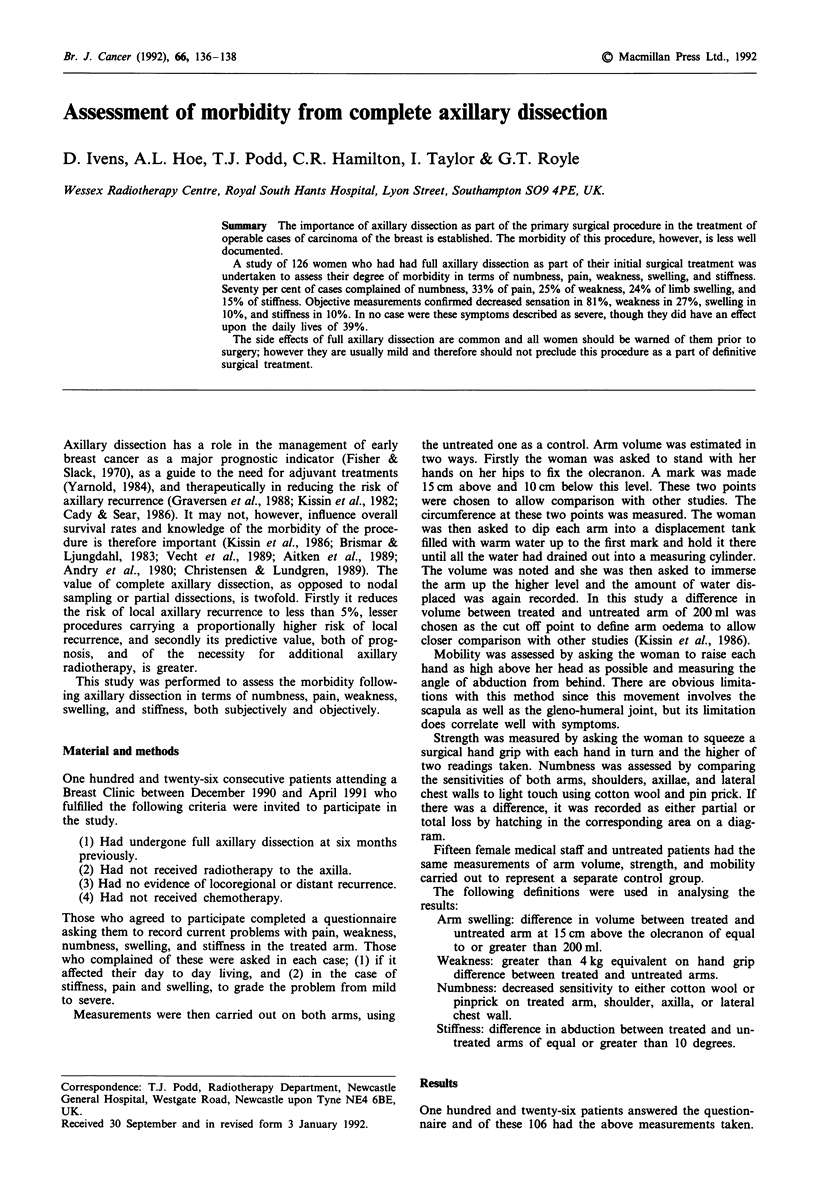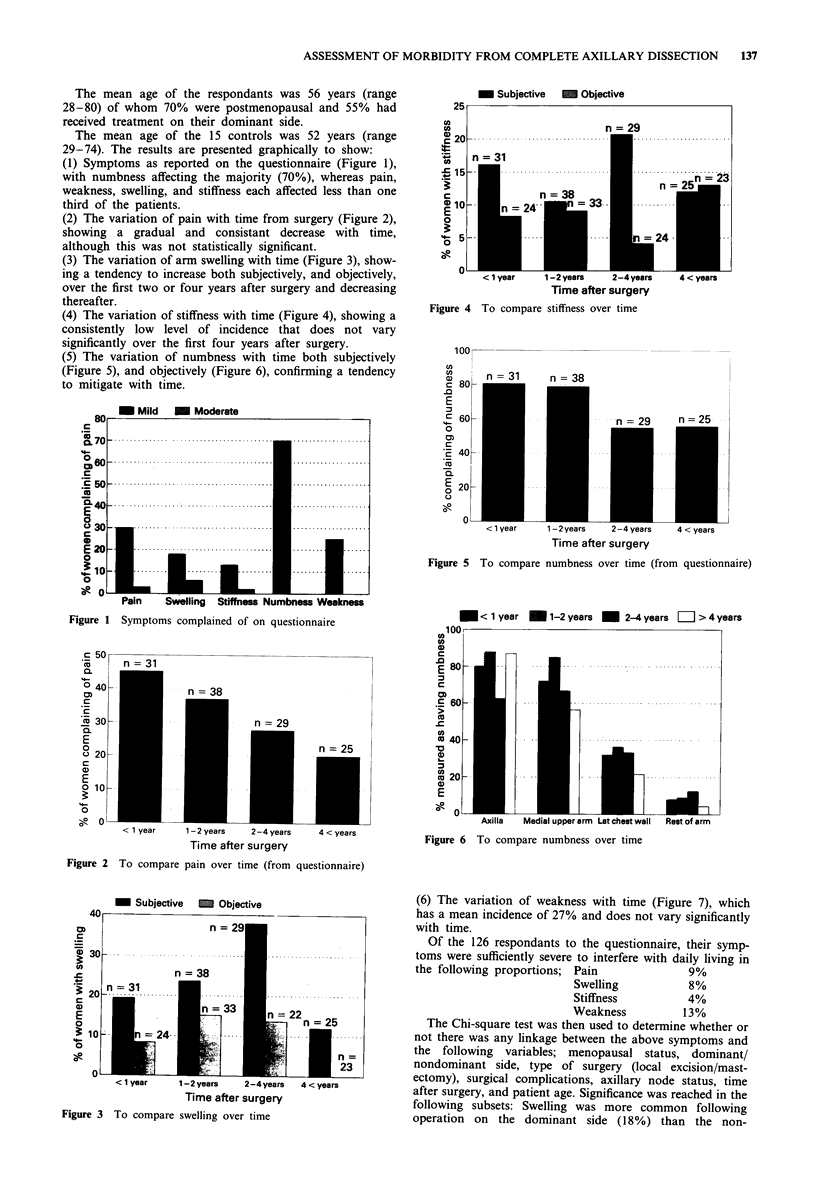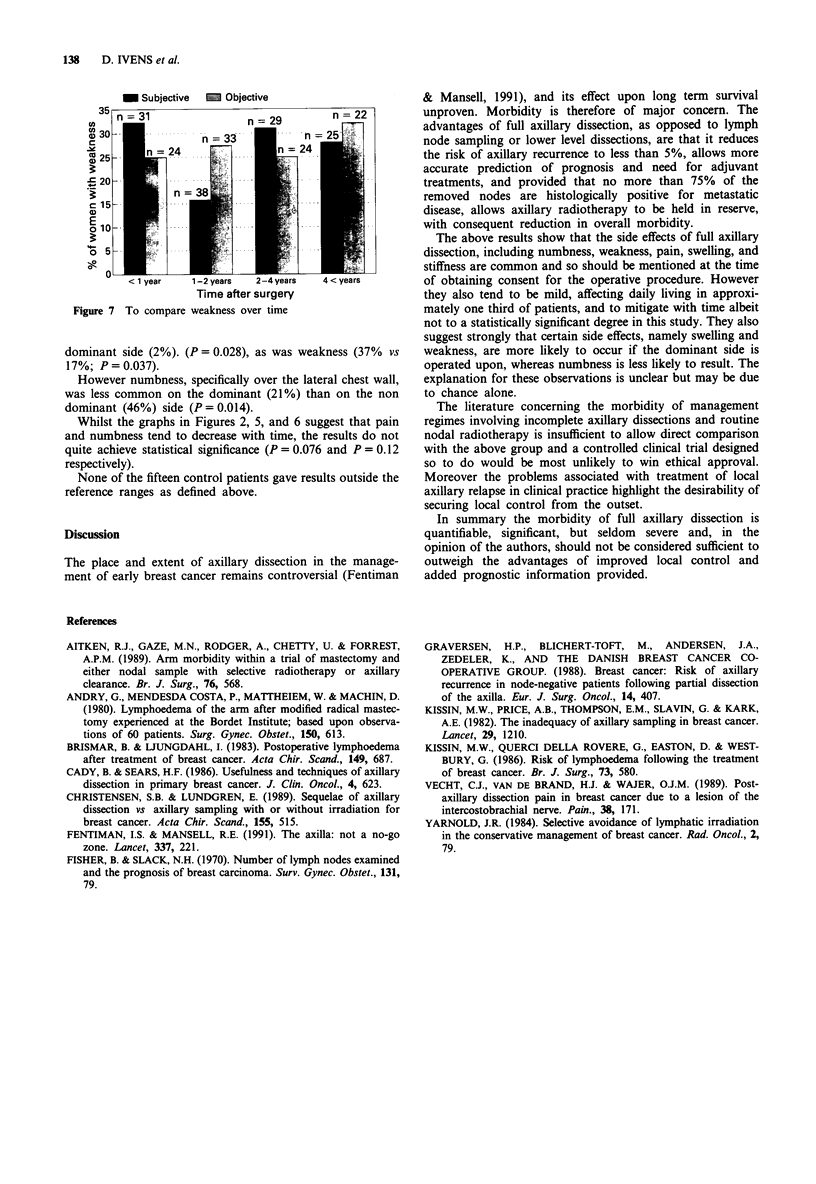# Assessment of morbidity from complete axillary dissection.

**DOI:** 10.1038/bjc.1992.230

**Published:** 1992-07

**Authors:** D. Ivens, A. L. Hoe, T. J. Podd, C. R. Hamilton, I. Taylor, G. T. Royle

**Affiliations:** Wessex Radiotherapy Centre, Royal South Hants Hospital, Southampton, UK.

## Abstract

The importance of axillary dissection as part of the primary surgical procedure in the treatment of operable cases of carcinoma of the breast is established. The morbidity of this procedure, however, is less well documented. A study of 126 women who had had full axillary dissection as part of their initial surgical treatment was undertaken to assess their degree of morbidity in terms of numbness, pain, weakness, swelling, and stiffness. Seventy per cent of cases complained of numbness, 33% of pain, 25% of weakness, 24% of limb swelling, and 15% of stiffness. Objective measurements confirmed decreased sensation in 81%, weakness in 27%, swelling in 10%, and stiffness in 10%. In no case were these symptoms described as severe, though they did have an effect upon the daily lives of 39%. The side effects of full axillary dissection are common and all women should be warned of them prior to surgery; however they are usually mild and therefore should not preclude this procedure as a part of definitive surgical treatment.


*Br. J. Cancer* (1992), **66**, 136–138 © Macmillan Press Ltd., 1992

### Assessment of morbidity from complete axillary dissection

D. Ivens, A.L. Hoe, T.J. Podd, C.R. Hamilton, I. Taylor & G.T. Royle

*Wessex Radiotherapy Centre, Royal South Hants Hospital, Lyon Street, Southampton SO9 4PE, UK.*

**Summary** The importance of axillary dissection as part of the primary surgical procedure in the treatment of operable cases of carcinoma of the breast is established. The morbidity of this procedure, however, is less well documented.

A study of 126 women who had had full axillary dissection as part of their initial surgical treatment was undertaken to assess their degree of morbidity in terms of numbness, pain, weakness, swelling, and stiffness. Seventy per cent of cases complained of numbness, 33% of pain, 25% of weakness, 24% of limb swelling, and 15% of stiffness. Objective measurements confirmed decreased sensation in 81%, weakness in 27%, swelling in 10%, and stiffness in 10%. In no case were these symptoms described as severe, though they did have an effect upon the daily lives of 39%.

The side effects of full axillary dissection are common and all women should be warned of them prior to surgery; however they are usually mild and therefore should not preclude this procedure as a part of definitive surgical treatment.

Axillary dissection has a role in the management of early breast cancer as a major prognostic indicator (Fisher & Slack, 1970), as a guide to the need for adjuvant treatments (Yarnold, 1984), and therapeutically in reducing the risk of axillary recurrence (Graversen *et al.*, 1988; Kissin *et al.*, 1982; Cady & Sear, 1986). It may not, however, influence overall survival rates and knowledge of the morbidity of the procedure is therefore important (Kissin *et al.*, 1986; Brismar & Ljungdahl, 1983; Vecht *et al.*, 1989; Aitken *et al.*, 1989; Andry *et al.*, 1980; Christensen & Lundgren, 1989). The value of complete axillary dissection, as opposed to nodal sampling or partial dissections, is twofold. Firstly it reduces the risk of local axillary recurrence to less than 5%, lesser procedures carrying a proportionally higher risk of local recurrence, and secondly its predictive value, both of prognosis, and of the necessity for additional axillary radiotherapy, is greater.

This study was performed to assess the morbidity following axillary dissection in terms of numbness, pain, weakness, swelling, and stiffness, both subjectively and objectively.

### Material and methods

One hundred and twenty-six consecutive patients attending a Breast Clinic between December 1990 and April 1991 who fulfilled the following criteria were invited to participate in the study.

(1) Had undergone full axillary dissection at six months previously.
(2) Had not received radiotherapy to the axilla.
(3) Had no evidence of locoregional or distant recurrence.
(4) Had not received chemotherapy.

Those who agreed to participate completed a questionnaire asking them to record current problems with pain, weakness, numbness, swelling, and stiffness in the treated arm. Those who complained of these were asked in each case; (1) if it affected their day to day living, and (2) in the case of stiffness, pain and swelling, to grade the problem from mild to severe.

Measurements were then carried out on both arms, using the untreated one as a control. Arm volume was estimated in two ways. Firstly the woman was asked to stand with her hands on her hips to fix the olecranon. A mark was made 15 cm above and 10 cm below this level. These two points were chosen to allow comparison with other studies. The circumference at these two points was measured. The woman was then asked to dip each arm into a displacement tank filled with warm water up to the first mark and hold it there until all the water had drained out into a measuring cylinder. The volume was noted and she was then asked to immerse the arm up the higher level and the amount of water displaced was again recorded. In this study a difference in volume between treated and untreated arm of 200 ml was chosen as the cut off point to define arm oedema to allow closer comparison with other studies (Kissin *et al.*, 1986).

Mobility was assessed by asking the woman to raise each hand as high above her head as possible and measuring the angle of abduction from behind. There are obvious limitations with this method since this movement involves the scapula as well as the gleno-humeral joint, but its limitation does correlate well with symptoms.

Strength was measured by asking the woman to squeeze a surgical hand grip with each hand in turn and the higher of two readings taken. Numbness was assessed by comparing the sensitivities of both arms, shoulders, axillae, and lateral chest walls to light touch using cotton wool and pin prick. If there was a difference, it was recorded as either partial or total loss by hatching in the corresponding area on a diagram.

Fifteen female medical staff and untreated patients had the same measurements of arm volume, strength, and mobility carried out to represent a separate control group.

The following definitions were used in analysing the results:

Arm swelling: difference in volume between treated and untreated arm at 15 cm above the olecranon of equal to or greater than 200 ml.

Weakness: greater than 4 kg equivalent on hand grip difference between treated and untreated arms.

Numbness: decreased sensitivity to either cotton wool or pinprick on treated arm, shoulder, axilla, or lateral chest wall.

Stiffness: difference in abduction between treated and untreated arms of equal or greater than 10 degrees.

### Results

One hundred and twenty-six patients answered the questionnaire and of these 106 had the above measurements taken.

Correspondence: T.J. Podd, Radiotherapy Department, Newcastle General Hospital, Westgate Road, Newcastle upon Tyne NE4 6BE, UK.
Received 30 September and in revised form 3 January 1992.

The mean age of the respondants was 56 years (range 28–80) of whom 70% were postmenopausal and 55% had received treatment on their dominant side.

The mean age of the 15 controls was 52 years (range 29–74). The results are presented graphically to show:

(1) Symptoms as reported on the questionnaire (Figure 1), with numbness affecting the majority (70%), whereas pain, weakness, swelling, and stiffness each affected less than one third of the patients.

(2) The variation of pain with time from surgery (Figure 2), showing a gradual and consistant decrease with time, although this was not statistically significant.

(3) The variation of arm swelling with time (Figure 3), showing a tendency to increase both subjectively, and objectively, over the first two or four years after surgery and decreasing thereafter.

(4) The variation of stiffness with time (Figure 4), showing a consistently low level of incidence that does not vary significantly over the first four years after surgery.

(5) The variation of numbness with time both subjectively (Figure 5), and objectively (Figure 6), confirming a tendency to mitigate with time.

**Figure 1** Symptoms complained of on questionnaire

**Figure 2** To compare pain over time (from questionnaire)

**Figure 3** To compare swelling over time

**Figure 4** To compare stiffness over time

**Figure 5** To compare numbness over time (from questionnaire)

**Figure 6** To compare numbness over time

(6) The variation of weakness with time (Figure 7), which has a mean incidence of 27% and does not vary significantly with time.

Of the 126 respondants to the questionnaire, their symptoms were sufficiently severe to interfere with daily living in the following proportions;

| | |
|---|---|
| Pain | 9% |
| Swelling | 8% |
| Stiffness | 4% |
| Weakness | 13% |

The Chi-square test was then used to determine whether or not there was any linkage between the above symptoms and the following variables; menopausal status, dominant/nondominant side, type of surgery (local excision/mastectomy), surgical complications, axillary node status, time after surgery, and patient age. Significance was reached in the following subsets: Swelling was more common following operation on the dominant side (18%) than the non-

**Figure 7** To compare weakness over time dominant side (2%). ($P = 0.028$), as was weakness (37% *vs* 17%; $P = 0.037$).

However numbness, specifically over the lateral chest wall, was less common on the dominant (21%) than on the non dominant (46%) side ($P = 0.014$).

Whilst the graphs in Figures 2, 5, and 6 suggest that pain and numbness tend to decrease with time, the results do not quite achieve statistical significance ($P = 0.076$ and $P = 0.12$ respectively).

None of the fifteen control patients gave results outside the reference ranges as defined above.

### Discussion

The place and extent of axillary dissection in the management of early breast cancer remains controversial (Fentiman & Mansell, 1991), and its effect upon long term survival unproven. Morbidity is therefore of major concern. The advantages of full axillary dissection, as opposed to lymph node sampling or lower level dissections, are that it reduces the risk of axillary recurrence to less than 5%, allows more accurate prediction of prognosis and need for adjuvant treatments, and provided that no more than 75% of the removed nodes are histologically positive for metastatic disease, allows axillary radiotherapy to be held in reserve, with consequent reduction in overall morbidity.

The above results show that the side effects of full axillary dissection, including numbness, weakness, pain, swelling, and stiffness are common and so should be mentioned at the time of obtaining consent for the operative procedure. However they also tend to be mild, affecting daily living in approximately one third of patients, and to mitigate with time albeit not to a statistically significant degree in this study. They also suggest strongly that certain side effects, namely swelling and weakness, are more likely to occur if the dominant side is operated upon, whereas numbness is less likely to result. The explanation for these observations is unclear but may be due to chance alone.

The literature concerning the morbidity of management regimes involving incomplete axillary dissections and routine nodal radiotherapy is insufficient to allow direct comparison with the above group and a controlled clinical trial designed so to do would be most unlikely to win ethical approval. Moreover the problems associated with treatment of local axillary relapse in clinical practice highlight the desirability of securing local control from the outset.

In summary the morbidity of full axillary dissection is quantifiable, significant, but seldom severe and, in the opinion of the authors, should not be considered sufficient to outweigh the advantages of improved local control and added prognostic information provided.

### References

AITKEN, R.J., GAZE, M.N., RODGER, A., CHETTY, U. & FORREST, A.P.M. (1989). Arm morbidity within a trial of mastectomy and either nodal sample with selective radiotherapy or axillary clearance. *Br. J. Surg.*, **76**, 568.

ANDRY, G., MENDESDA COSTA, P., MATTHEIEM, W. & MACHIN, D. (1980). Lymphoedema of the arm after modified radical mastectomy experienced at the Bordet Institute; based upon observations of 60 patients. *Surg. Gynec. Obstet.*, **150**, 613.

BRISMAR, B. & LJUNGDAHL, I. (1983). Postoperative lymphoedema after treatment of breast cancer. *Acta Chir. Scand.*, **149**, 687.

CADY, B. & SEARS, H.F. (1986). Usefulness and techniques of axillary dissection in primary breast cancer. *J. Clin. Oncol.*, **4**, 623.

CHRISTENSEN, S.B. & LUNDGREN, E. (1989). Sequelae of axillary dissection *vs* axillary sampling with or without irradiation for breast cancer. *Acta Chir. Scand.*, **155**, 515.

FENTIMAN, I.S. & MANSELL, R.E. (1991). The axilla: not a no-go zone. *Lancet*, **337**, 221.

FISHER, B. & SLACK, N.H. (1970). Number of lymph nodes examined and the prognosis of breast carcinoma. *Surv. Gynec. Obstet.*, **131**, 79.

GRAVERSEN, H.P., BLICHERT-TOFT, M., ANDERSEN, J.A., ZEDELER, K., AND THE DANISH BREAST CANCER CO-OPERATIVE GROUP. (1988). Breast cancer: Risk of axillary recurrence in node-negative patients following partial dissection of the axilla. *Eur. J. Surg. Oncol.*, **14**, 407.

KISSIN, M.W., PRICE, A.B., THOMPSON, E.M., SLAVIN, G. & KARK, A.E. (1982). The inadequacy of axillary sampling in breast cancer. *Lancet*, **29**, 1210.

KISSIN, M.W., QUERCI DELLA ROVERE, G., EASTON, D. & WESTBURY, G. (1986). Risk of lymphoedema following the treatment of breast cancer. *Br. J. Surg.*, **73**, 580.

VECHT, C.J., VAN DE BRAND, H.J. & WAJER, O.J.M. (1989). Post-axillary dissection pain in breast cancer due to a lesion of the intercostobrachial nerve. *Pain.*, **38**, 171.

YARNOLD, J.R. (1984). Selective avoidance of lymphatic irradiation in the conservative management of breast cancer. *Rad. Oncol.*, **2**, 79.